# Regulatory T Cell-Related Gene Biomarkers in the Deterioration of Atherosclerosis

**DOI:** 10.3389/fcvm.2021.661709

**Published:** 2021-05-20

**Authors:** Meng Xia, Qingmeng Wu, Pengfei Chen, Cheng Qian

**Affiliations:** ^1^Department of Cardiology, The Affiliated Hospital of Southwest Medical University, Luzhou, China; ^2^Healthcare-Associated Infections Control Center, The Affiliated Chinese Medicine Hospital of Southwest Medical University, Luzhou, China; ^3^Department of Gastroenterology, The Central Hospital of Enshi Tujia and Miao Autonomous Prefecture, Enshi, China

**Keywords:** regulatory T cells, atherosclerosis, progression, biological network, genes

## Abstract

**Background:** Regulatory T cells (Tregs) have shown to be protective against the development of atherosclerosis, a major pathological cause for cardiovascular events. Here, we aim to explore the roles of Tregs-related genes in atherosclerosis deterioration.

**Methods and Results:** We downloaded the gene expression profile of 29 atherosclerotic samples from the Gene Expression Omnibus database with an accession number of GSE28829. The abundance of Tregs estimated by the CIBERSORT algorithm was negatively correlated with the atherosclerotic stage. Using the limma test and correlation analysis, a total of 159 differentially expressed Tregs-related genes (DETregRGs) between early and advanced atherosclerotic plaques were documented. Functional annotation analysis using the DAVID tool indicated that the DETregRGs were mainly enriched in inflammatory responses, immune-related mechanisms, and pathways such as complement and coagulation cascades, platelet activation, leukocyte trans-endothelial migration, vascular smooth muscle contraction, and so on. A protein-protein interaction network of the DETregRGs was then constructed, and five hub genes (*PTPRC, C3AR1, CD53, TLR2*, and *CCR1*) were derived from the network with node degrees ≥20. The expression patterns of these hub DETregRGs were further validated in several independent datasets. Finally, a single sample scoring method was used to build a gene signature for the five DETregRGs, which could distinguish patients with myocardial infarction from those with stable coronary disease.

**Conclusion:** The results of this study will improve our understanding about the Tregs-associated molecular mechanisms in the progression of atherosclerosis and facilitate the discovery of novel biomarkers for acute cardiovascular events.

## Introduction

Despite significant advances in treatment, cardiovascular disease (CVD) remains a major public health issue in both developed and developing regions, accounting for nearly 18.6 million deaths worldwide in the year 2019 (1). The primary underlying cause of CVD is atherosclerosis, a chronic vascular disorder characterized by endothelial dysfunction, abundant lipid accumulation, dysregulation of vascular smooth muscle (VSM) cells, immunoinflammatory activation, and formation of atherosclerotic plaques (2). Along with atherosclerosis deterioration, the plaques may become unstable and vulnerable to rupture, which subsequently leads to thrombosis in diseased vessels with acute clinical manifestations such as myocardial infarction (MI) and stroke (3, 4). These complications of atherosclerosis contribute greatly to the severe burden of mortality and disability in patients suffering from CVD (5). Therefore, discovery of novel biomarkers related to atherosclerosis deterioration may improve the clinical management of CVD patients.

There is growing evidence that autoimmunity may play critical roles in the development of atherosclerosis (6). Atherosclerotic plaques are populated by cells involved in the innate and adaptive immune system (7), of which regulatory T cells (Tregs) have received growing interests in recent researches. In addition to suppressing the immune response mediated by effector T and B cells, Tregs have been shown to secrete inhibitory cytokines, inhibit macrophage inflammation, and modulate cholesterol metabolism and foam cell formation (8). Owing to these biological proprieties, Tregs are considered to be an atheroprotective component that functions importantly in maintaining immune homeostasis in plaques. In human atherosclerosis, the decreased number of Tregs in atherosclerotic plaques was reportedly linked to plaque vulnerability (9) and rupture (10), while restoring the abundance of Tregs could dampen atherosclerotic progression (11). Although the negative association between circulating Tregs abundance and CVD has not been well-established (12, 13), targeting Tregs is increasingly considered a promising strategy to improve the outcome of CVD patients.

The development of microarrays and RNA-sequencing has accelerated the discovery of novel diagnostic or therapeutic targets for CVD. With these technologies, previous studies have found some genetic biomarkers in the deterioration of atherosclerosis (14, 15); however, relationships between Tregs and these biomarkers and molecular mechanisms by which Tregs suppress atherosclerosis still remain unknown. In this study, we quantified the Tregs compositions of atherosclerotic samples using the CIBERSORT deconvolution method. The differentially expressed Tregs-related genes (DETregRGs) between early and advanced atherosclerotic plaques were obtained using the limma test and correlation analysis. The potential biological functions and pathways of the DETregRGs were then explored, followed by the construction of a protein-protein interaction (PPI) network to identify hub genes, which were further validated in independent atherosclerotic samples. Finally, the hub DETregRGs were integrated to form a multigene signature for the diagnosis of acute ischemic events such as MI. The work flow for our study is described in Supplementary Figure 1. The expected results may facilitate our knowledge about the Tregs-associated molecular mechanisms in atherosclerosis progression and provide potential biomarkers for acute cardiovascular events.

## Methods and Materials

### Data Sources

We downloaded the human microarray data of GSE28829 from the Gene Expression Omnibus (GEO, https://www.ncbi.nlm.nih.gov/geo/) database. This dataset profiled the gene expressions of 16 advanced and 13 early carotid atherosclerotic plaques based on the Affymetrix HG-U133 Plus 2.0 array (16). Before further analyses, the raw transcriptomic data were processed with normalization and log2 transformation using a robust multichip average algorithm (17).

### Evaluation of Immune Cells in Atherosclerotic Plaques

CIBERSORT is a deconvolution approach for characterizing cell compositions of bulk tissues based on their gene expression profiles (18). To acquire the abundance of immune cells in atherosclerotic plaques, we applied the CIBERSORT algorithm with 100 permutations to the GSE28829 dataset, using the LM22 matrix as reference. With the Monte Carlo Sampling method, CIBERSORT outputs a deconvolution *p*-value for each sample to measure the reliability of the results. In this study, we retained only the atherosclerotic samples with *p* < 0.05 to analyze the fractions of immune cells. The Wilcoxon rank-sum test was used to compare the abundance of each cell type between plaques in early and advanced stages.

### Identification of DETregRGs

The differentially expressed genes (DEGs) between advanced and early atherosclerotic plaques were obtained using the “limma” package in R. The genes with |fold change|>1.5 or *p* < 0.01 were considered differentially expressed. Then, Pearson correlation analysis was performed to identify the genes associated with the abundance of Tregs. The DEGs with Pearson correlation coefficients (PCC) >0.6 were treated as DETregRGs.

### Functional Enrichment Analysis

The Database for Annotation, Visualization, and Integrated Discovery (DAVID, https://david.ncifcrf.gov/) is a web-accessible program designed for users to explore the biological meaning behind a cluster of genes (19). Therefore, to understand the potential functions of DETregRGs, enrichment analyses for Gene Ontology (GO) biological processes and Kyoto Encyclopedia of Genes and Genomes (KEGG) pathway were conducted using the DAVID 6.8 online tool. The significance threshold for enrichment analyses was set as *p* < 0.05 and gene count >3.

### Construction of the PPI Network

The Search Tool for the Retrieval of Interacting Genes (STRING, https://string-db.org) is a biological resource that provides systematic screens of human protein interactions (20). In this study, we uploaded the DETregRGs list into the STRING database to identify significant PPIs with a combined score >0.4. The PPI network was built and then visualized using Cytoscape 3.6.0 software. Nodes with a connectivity degree ≥20 in the network were selected as hub DETregRGs.

### Validation of Hub Genes in Atherosclerosis Deterioration

To view the association between hub DETregRGs and atherosclerosis progression, we conducted unsupervised hierarchical clustering in the GSE28829 dataset using the “Pheatmap” package in R. Then, the expression patterns of hub DETregRGs across disease stages were validated in two independent datasets comparing stable vs. unstable plaques, including GSE120521 (*n* = 8) (21) and E-TABM-190 (*n* = 11) (22). We also used the GSE163154 (*n* = 43) (23) dataset to further confirm the expressions of hub genes in plaques with or without intraplaque hemorrhage, a crucial feature of vulnerable atherosclerotic lesions (24). The detailed information for these datasets was demonstrated in Supplementary Table 1.

### Construction of Hub DETregRGs Signature

The hub DETregRGs were integrated to form a multigene signature using a single sample scoring algorithm called “singscore” (25). This method generates scores that are stable across a range of sample sizes and numbers of measured genes. In this study, the scores against the hub DETregRGs set (termed “TregRG score”) were calculated for the peripheral blood samples collected at admission from coronary artery disease (CAD) patients in datasets of GSE59867 (*n* = 157) (26) and GSE62646 (*n* = 42) (27). Receiver operation characteristic (ROC) curve analyses were performed to evaluate the diagnostic performance of the TregRG score for acute ischemic events, and the area under the curve (AUC) was computed using “pROC” package in R. An equal number of genes were randomly selected to generate a random score for background comparison using the Bootstrap method.

## Results

### DETregRGs in Atherosclerosis Progression

**Figure 1:**
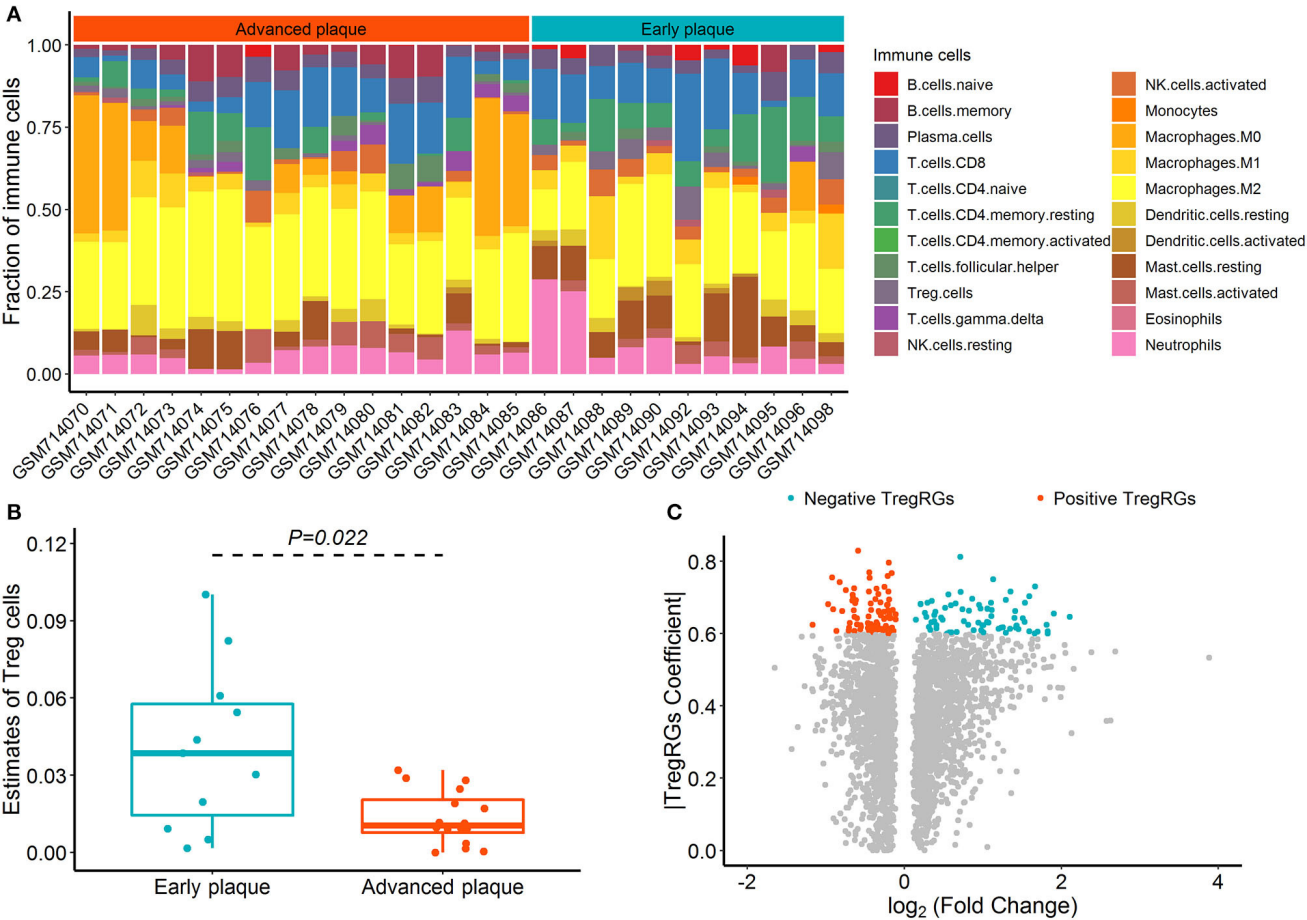
Identification of DETregRGs in advanced and early atherosclerotic plaques. **(A)** Abundance of 22 immune cells across 27 samples estimated by the CIBERSORT algorithm. **(B)** Fraction of Tregs between 11 early and 16 advanced plaque samples. **(C)** Volcano plot for the differentially expressed genes between 13 early and 16 advanced plaque samples. DETregRGs, differentially expressed Tregs-related genes; Tregs, regulatory T cells; TregRGs, Tregs-related genes.

The CIBERSORT deconvolution algorithm was exploited to access the immune cell compositions in the GSE28829 dataset, and two samples with deconvolution *p* ≥ 0.05 were excluded (Supplementary Table 2). Figure 1A summarizes the results obtained from the remaining 27 plaque samples. Compared with advanced atherosclerotic plaques, the plaques in the early stage exhibited a higher infiltration of Tregs (Figure 1B; *p* = 0.022). Using the limma test, we obtained a total of 2,892 DEGs between 16 advanced and 13 early atherosclerotic samples. Of these DEGs, 159 were highly correlated (PCC > 0.6) with the abundance of Tregs and were identified as DETregRGs (Figure 1C; Supplementary Table 3). In addition to Tregs, there were eight immune cell types also exhibiting different proportions between early and advanced plaques, including Macrophages M2, Macrophages M0, memory B cells, gamma delta T cells, naive B cells, activated dendritic cells, resting mast cells, and resting memory CD4^+^ T cells. However, none of these immune cells showed PCC values >0.6 with more than 20% of DETregRGs (Supplementary Table 4), suggesting that the identified genes were associated predominately with Tregs in the progression of atherosclerosis.

### Functional Enrichment for DETregRGs

The list of DETregRGs was uploaded into the DAVID 6.8 web page for functional analysis. These genes were mainly involved in GO-biological processes such as inflammatory response, lipopolysaccharide-mediated signaling, cell surface receptor signaling, MyD88-dependent toll-like receptor (TLR) signaling, and chemotaxis. Moreover, KEGG pathway enrichment analysis showed that the signaling pathways associated with DETregRGs included complement and coagulation cascades, platelet activation, chemokine signaling pathway, leukocyte trans-endothelial migration, and VSM contraction (Table 1).

**Table 1:** The significant GO biological processes and KEGG pathways enriched by DETregRGs.

**ID**	**Term**	***p*-value**	**Count**	**Gene symbol**
**GO-biological processes (top 5)**
GO:0006954	Inflammatory response	<0.001	13	*LYN, PTGIR, F11R, C3AR1*.
GO:0031663	Lipopolysaccharide-mediated signaling	<0.001	5	*LYN, CCL5, CD14, TLR2*.
GO:0007166	Cell surface receptor signaling	0.002	9	*CD53, CCR1, MARCO, PTPRC*.
GO:0002755	MyD88-dependent toll-like receptor signaling	0.002	4	*CD14, TLR5, UBA52*, and *TLR2*
GO:0006935	Chemotaxis	0.003	6	*CCR1, CCL5, RAC2, C3AR1*.
**KEGG pathway (top 5)**
hsa04610	Complement and coagulation cascades	<0.001	6	*C3AR1, C1QB, C1QA, CFH*.
hsa04611	Platelet activation	0.002	7	*LYN, PTGIR, ITPR2, VAMP8*.
hsa04062	Chemokine signaling pathway	0.002	8	*CCR1, HCK, CCL5, CXCR4*.
hsa04670	Leukocyte trans-endothelial migration	0.006	6	*CXCR4, F11R, CTNNA1, RAC2*.
hsa04270	Vascular smooth muscle contraction	0.006	6	*PTGIR, NPR1, PLA2G2A, ITPR2*.

*DETregRGs, differentially expressed regulatory T cell-related genes; GO, gene ontology; KEGG, Kyoto Encyclopedia of Genes and Genomes*.

### PPI Network Analysis

**Figure 2:**
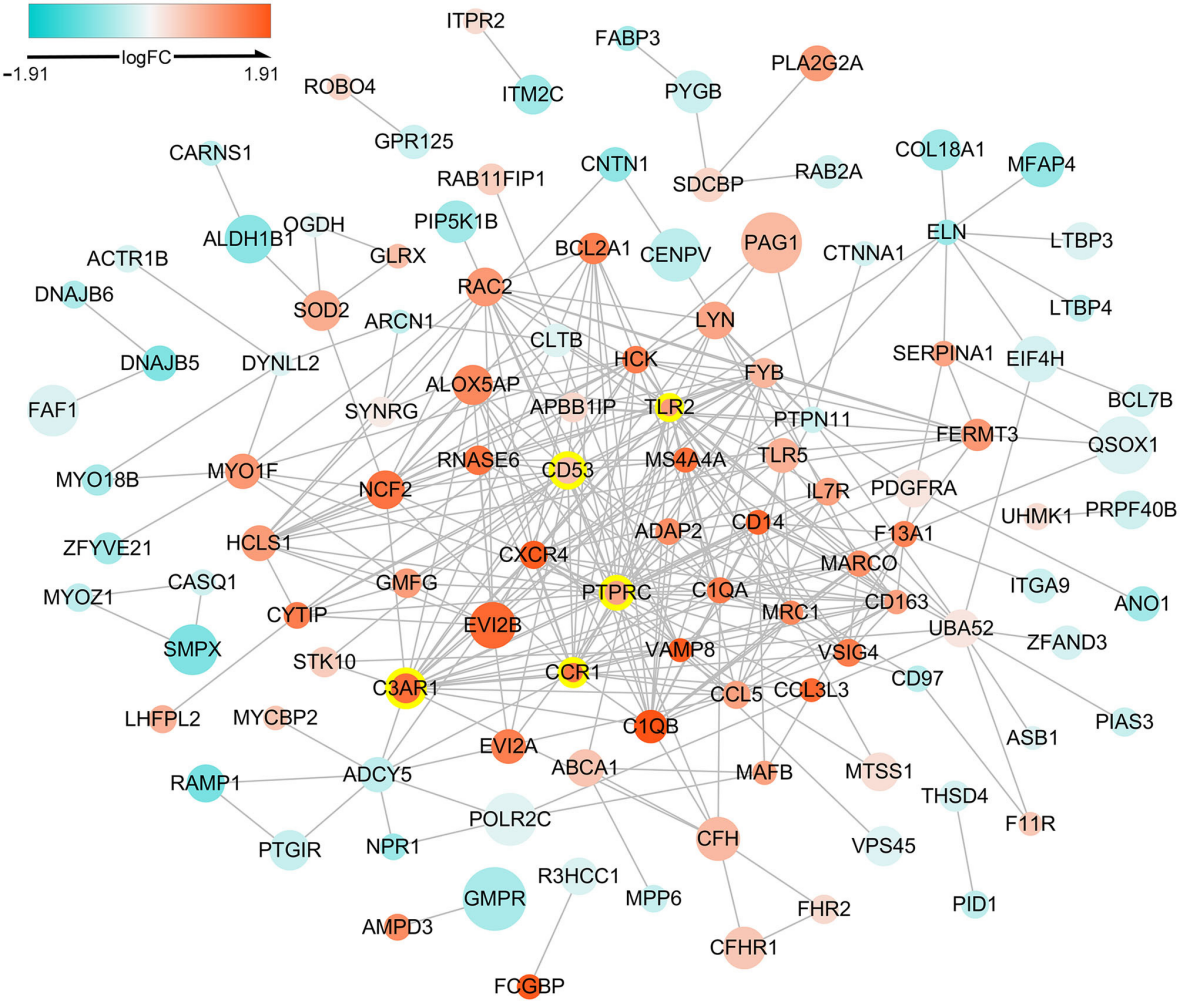
Protein-protein interaction network for the DETregRGs. The node size stands for the Pearson correlation coefficient of a certain gene with Tregs abundance. The nodes with yellow outlines represent hub genes. DETregRGs, differentially expressed Tregs-related genes; FC, fold change.

The PPI network of DETregRGs constructed using the STRING database was a scale-free network (Correlation = 0.979, *R*^2^ = 0.713), which contained 113 nodes and 323 edges (Figure 2). The genes with connective degrees ≥20 were screened out as hub DETregRGs in the network, including *PTPRC* (degree = 32), *C3AR1* (degree = 22), *CD53* (degree = 22), *TLR2* (degree = 21), and *CCR1* (degree = 20). These hub genes were all up-regulated in advanced as compared with early atherosclerotic tissues.

### Validation of Hub DETregRGs in Atherosclerotic Lesions

**Figure 3:**
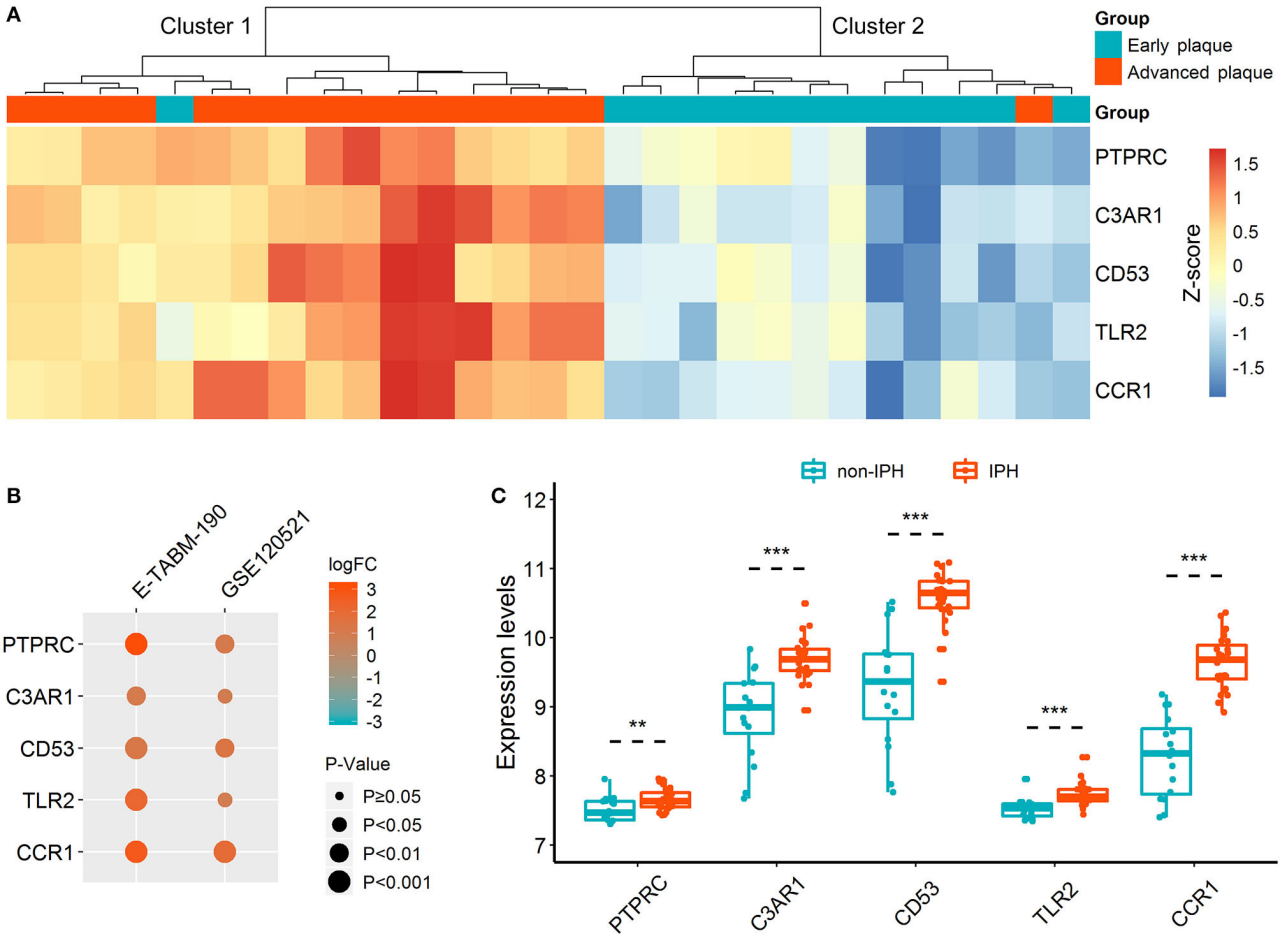
Expression patterns of hub DETregRGs between advanced and early atherosclerotic plaques. **(A)** The heat map for unsupervised hierarchical clustering of the five hub DETregRGs in GSE28829 dataset. **(B)** Validation of the hub DETregRGs between stable and unstable atherosclerotic plaques. **(C)** Validation of the hub DETregRGs between atherosclerosis with and without IPH. **p < 0.01, compared with the non-IPH group; ***p < 0.001, compared with the non-IPH group. DETregRGs, differentially expressed Tregs-related genes; FC, fold change; IPH, intraplaque hemorrhage.

Unsupervised hierarchical clustering was performed according to the expressions of hub DETregRGs in GSE28829 dataset. In Figure 3A, the samples were divided into two distinct clusters: the majority of advanced atherosclerotic samples were grouped into cluster 1 (15/16, 94%), and most control samples were grouped into cluster 2 (12/13, 92%). The results suggested that the five genes had a potential association with the disease status of atherosclerosis. We next validated the gene expression patterns in two independent datasets with a small sample size (GSE120521 and E-TABM-190) using the limma test. As shown in Figure 3B, all of the key DETregRGs were highly expressed in atherosclerotic plaques with unstable status. To further confirm this finding, the correlations of the five DETregRGs with intraplaque hemorrhage were examined in the GSE163154 dataset (*n* = 43). The results of a Wilcoxon rank-sum test demonstrated that these genes were all up-regulated in atherosclerotic plaques with as compared with those without intraplaque hemorrhage (Figure 3C; all *p* < 0.01).

### Clinical Significance of the Hub Gene Signature

**Figure 4:**
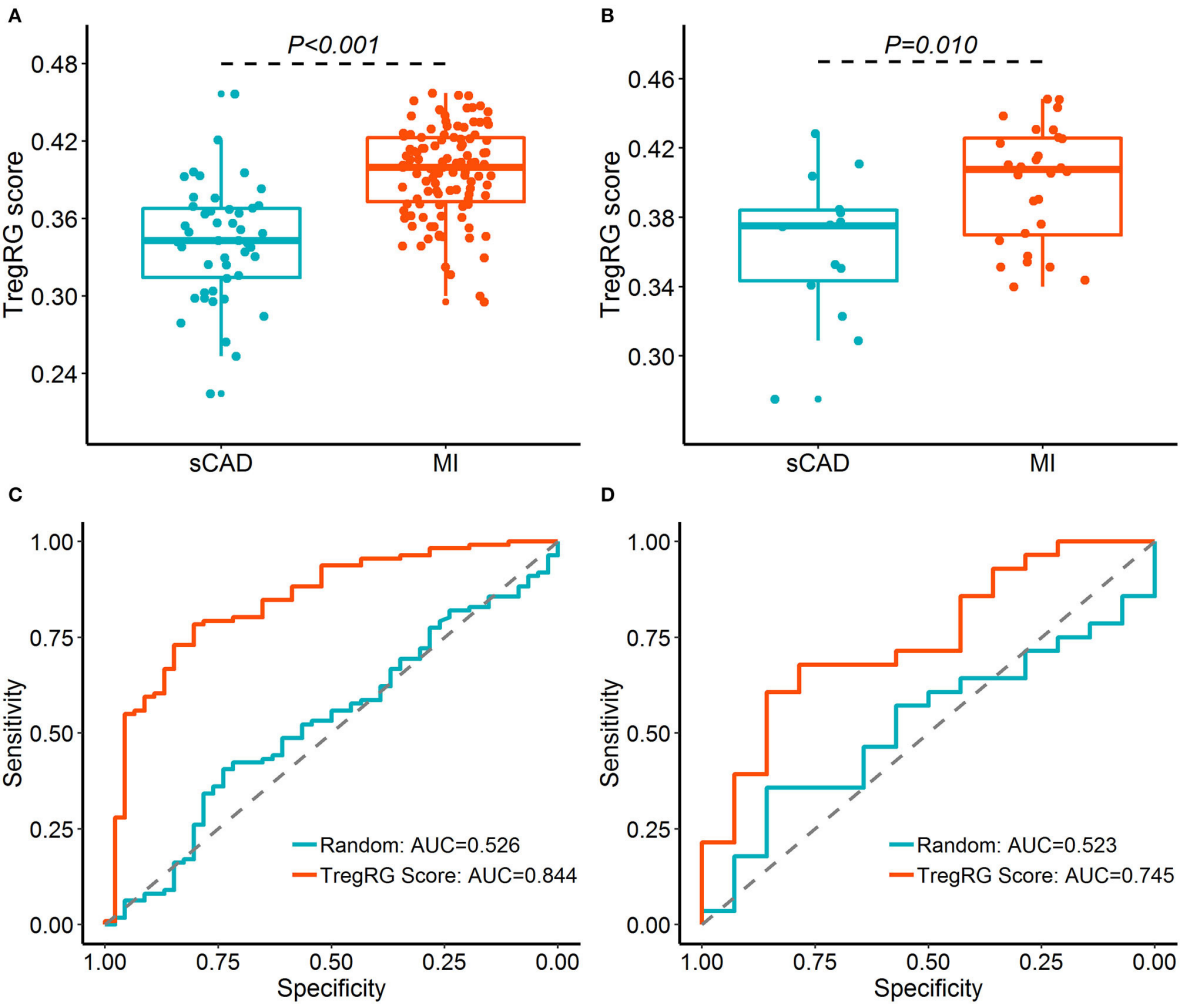
Diagnostic performance of the TregRG score for patients with MI. **(A,B)** TregRG scores between patients with sCAD and MI in GSE59867 and GSE62646 datasets, respectively. **(C,D)** Receiver operation characteristic curves of TregRG score for diagnosis of MI in GSE59867 and GSE62646 datasets, respectively. AUC, area under the curve; MI, myocardial infarction; sCAD, stable coronary artery disease.

Since atherosclerosis deterioration is a major contributor to acute cardiovascular events, we inferred that the hub DETregRGs may be helpful to identify patients with ischemic attacks such as MI. To test this hypothesis, the five DETregRGs were aggregated to calculate TregRG scores using the “singscore” algorithm, as described in the “Methods and Materials” section. In the datasets of GSE59867 and GSE62646, patients with MI showed significantly higher TregRG scores than those with stable CAD (Figures 4A,B). ROC analyses suggested that the AUC of TregRG score to discriminate MI patients was 0.844 in the GSE59867 dataset, which was higher than that of a random condition (Figure 4C; Bootstrap *p* < 0.001**)**. A similar result was achieved in the dataset of GSE62646 (Figure 4D; AUC = 0.745, Bootstrap *p* = 0.024).

## Discussion

Recent studies based on single-cell RNA-sequencing or cytometry have confirmed the decreased fractions of Tregs in atherosclerotic aortas and demonstrated the loss of immunosuppressive and atheroprotective function for this immune cell subset during the course of atherosclerosis (28). And in mouse models of plaque regression, an increase in Tregs was suggested as a common feature of attenuated inflammation and regressing atherosclerosis (29). However, the Tregs-related molecular mechanisms and genetic biomarkers in the progression of atherosclerosis still remain under-investigated. In our study, consistent with previous reports, the number of Tregs was decreased in atherosclerotic plaques in advanced vs. early stages. We then identified a total of 159 DETregRGs between the advanced and early samples, and these genes were mainly associated with inflammatory responses, immune-related mechanisms, and pathways such as complement and coagulation cascades, platelet activation, and VSM contraction. In the PPI network, *PTPRC, C3AR1, CD53, TLR2*, and *CCR1* were considered as hub genes based on their connective degrees. The expression patterns of these hub DETregRGs between different disease stages could be validated in independent datasets. More importantly, the hub gene signature constructed using the “singscore” algorithm was capable of discriminating between patients with MI and those with stable CAD.

The DETregRGs were found to be involved in some immunoinflammatory processes including lipopolysaccharide-mediated signaling and MyD88-dependent TLR signaling. The cellular responses to both exogenous and endogenous lipopolysaccharide are mediated by TLRs, which, it has been suggested, function importantly in not only atherosclerotic pathogenesis but plaque destabilization as well (30). As a crucial downstream target shared by most TLRs, the MyD88 signaling could enhance immunity and promote plaque growth and might regulate plaque stability in atherosclerosis (31). More importantly, MyD88-dependent TLR signaling was shown to be essential in the suppression of atherosclerosis mediated by Tregs (32). These highlighted the potential role of TLRs/MyD88 cascade in the Tregs response and progression of atherosclerosis.

Moreover, the DETregRGs were also enriched in many pathways related to plaque destabilization, such as complement and coagulation cascade, platelet activation, chemokine signaling, leukocyte migration across the endothelium, and VSM contraction. The complement and coagulation cascade is a complex biological process that links immune response to thrombotic status. The concurrent activation of the complement and coagulation system is evident in atherothrombosis, as reflected by the accumulation of coagulation factors (33) and complements such as C3a and C5a (34). Platelet activation is the cornerstone of atherothrombosis, which depends on many dysregulated factors, including endothelial dysfunction, oxidized lipoproteins, and immune response (35). Chemokine-induced leukocyte trans-endothelial migration has been accepted as a key feature of both early and late stage atherosclerosis (36), and reduction of leukocyte recruitment into plaques may be a promising alternative in the treatment of plaque vulnerability (37). VSM cells play pivotal roles in atherosclerotic plaque initiation, development and stability via phenotypic switching from “contractile” to “synthetic” state or other cell types (38). Previous studies have demonstrated that inhibition of the phenotypic switching of VSM cells could suppress atherosclerotic plaque growth and improve plaque stability (39, 40). Interestingly, we observed that the immunoinflammatory biological processes and atherosclerosis deterioration-related pathways shared some common genes, including *LYN, PTGIR, F11R*, and *C3AR1* (Table 1). These results indicated that the DETregRGs may be involved in the immune-inflammation-mediated progression of atherosclerosis.

The present study identified five hub DETregRGs from the PPI network, including *PTPRC, C3AR1, CD53, TLR2*, and *CCR1*. Among these genes, only *TLR2* has been directly correlated with atherosclerotic plaque evolution and destabilization. Indeed, *TLR2* expression was elevated in advanced human arterial lesions (41), and blocking TLR2 activity alleviates and stabilizes advanced atherosclerotic plaques in apolipoprotein-E-deficient mice by suppressing inflammatory cytokines and attenuating macrophage apoptosis (42). Both *PTPRC* and *CD53* are leukocyte antigens; the former serves as an essential regulator of the immune signaling of T and B cells (43), while the latter participates in the transduction of CD2-generated signals in T cells and natural killer cells (44). *C3AR1* is an orphan G protein-coupled receptor for C3a, a potent pro-inflammatory and chemotactic peptide during complement activation. A recent study showed that in apolipoprotein-E-deficient mice, silencing *C3AR1* might result in a more serious atherosclerotic burden and inflammatory response (45). As a binding receptor of C-C motif chemokine ligand 5, *CCR1* is expressed in various cell types such as macrophages, T lymphocytes, and Th1 cells. Braunersreuther et al. (46) observed that *CCR1* deficiency could exacerbate T cell recruitment and enhance atherosclerotic plaque development, while Zernecke et al. (47) observed no effects on plaque formation or cell content.

Although the roles of most hub DETregRGs have not been established in atherosclerosis, we found their expression patterns between advanced and early carotid plaques were consistent across independent datasets. Indeed, these genes have also been implicated in the development of other immune-inflammatory diseases such as asthma and rheumatoid arthritis (48, 49). The carotid and coronary arteries are the two most common systems afflicted by atherosclerosis. Despite the different anatomical locations, the two artery systems have been suggested to share a similar phenotypic pattern of atherosclerosis in plaque formation, luminal narrowing or obstruction, and arterial wall calcification (50). Therefore, the hub genes derived from carotid atherosclerosis deterioration may be related to the acute clinical phenotype of coronary plaques such as MI. To test this hypothesis, we constructed a multigene signature for the hub DETregRGs and found that this signature had strong performance in distinguishing patients with MI from those with stable CAD. Since the peripheral blood samples analyzed in our study were obtained at admission, the hub gene signature may be helpful for early risk profiling of atherosclerotic patients. However, due to the potential differences between transcriptome profiles in circulating blood cells and atherosclerotic plaque tissues, this finding requires further prospective validations.

Some limitations should be acknowledged in this study. Firstly, the abundance of Tregs was estimated by the CIBERSORT algorithm using the LM22 mixture as reference, which was derived from circulating leukocytes or *in vitro* manipulated cells, not from bulk tissues. Also, in long-standing chronic conditions such as atherosclerosis, some immune cell types may alter their gene expression patterns. Although CIBERSORT has been widely used to quantify cell fractions in complex tissues and has outperformed other available approaches in handling closely related cell subtypes, unknown mixture content, and noise (18), these transcriptional deviations may introduce some inaccuracy to the deconvolution results. Secondly, due to the cross-sectional design of the original datasets, it is infeasible to clarify the causality between DETregRGs expressions and Tregs abundance. Thirdly, the hub DETregRGs signature was investigated in datasets with no access to individual patients' characteristics; thus, we cannot adjust the ROC curve for traditional cardiovascular risk factors. A prospective cohort recruiting atherosclerotic patients is needed to confirm the prognostic value of hub DETregRGs. Last but not least, cellular and animal experiments are lacking in the current study. Although the expressions of hub DETregRGs were validated using *in silico* data, the detailed molecular mechanisms related to these genes remain largely unknown and should be revealed in future experiments.

In summary, this study provides a comprehensive view on the Tregs-related genes that may be associated with the disease stages of atherosclerosis. We have identified several molecular mechanisms potentially linking Tregs to atherosclerosis deterioration. In addition, five genes were considered to play critical roles in atherosclerotic plaque evolution and destabilization. These results may help us to better understand the functional mechanisms related to Tregs in atherosclerosis deterioration and provide candidate biomarkers for the diagnosis or treatment of acute cardiovascular attacks.

## Data Availability Statement

The original contributions presented in the study are included in the article/Supplementary Material, further inquiries can be directed to the corresponding author/s.

## Ethics Statement

Ethical review and approval was not required for the study on human participants in accordance with the local legislation and institutional requirements. Written informed consent for participation was not required for this study in accordance with the national legislation and the institutional requirements.

## Author Contributions

CQ conceived and designed the study. MX, QW, and PC performed the study and contributed to data management and analysis. MX wrote the paper, with key intellectual content revised by CQ and QW. All authors read and approved the final submission.

## Conflict of Interest

The authors declare that the research was conducted in the absence of any commercial or financial relationships that could be construed as a potential conflict of interest.
