# Supplementary material for: Regulatory T Cell-Related Gene Biomarkers in the Deterioration of Atherosclerosis
Source: Front Cardiovasc Med. 2021 May 20;8:661709. doi: 10.3389/fcvm.2021.661709 (PMC8172618; doi:10.3389/fcvm.2021.661709)
Supplement: Supplementary file 1 [file Data_Sheet_1.pdf]

Supplementary Figure 1. The flow chart of data analyses

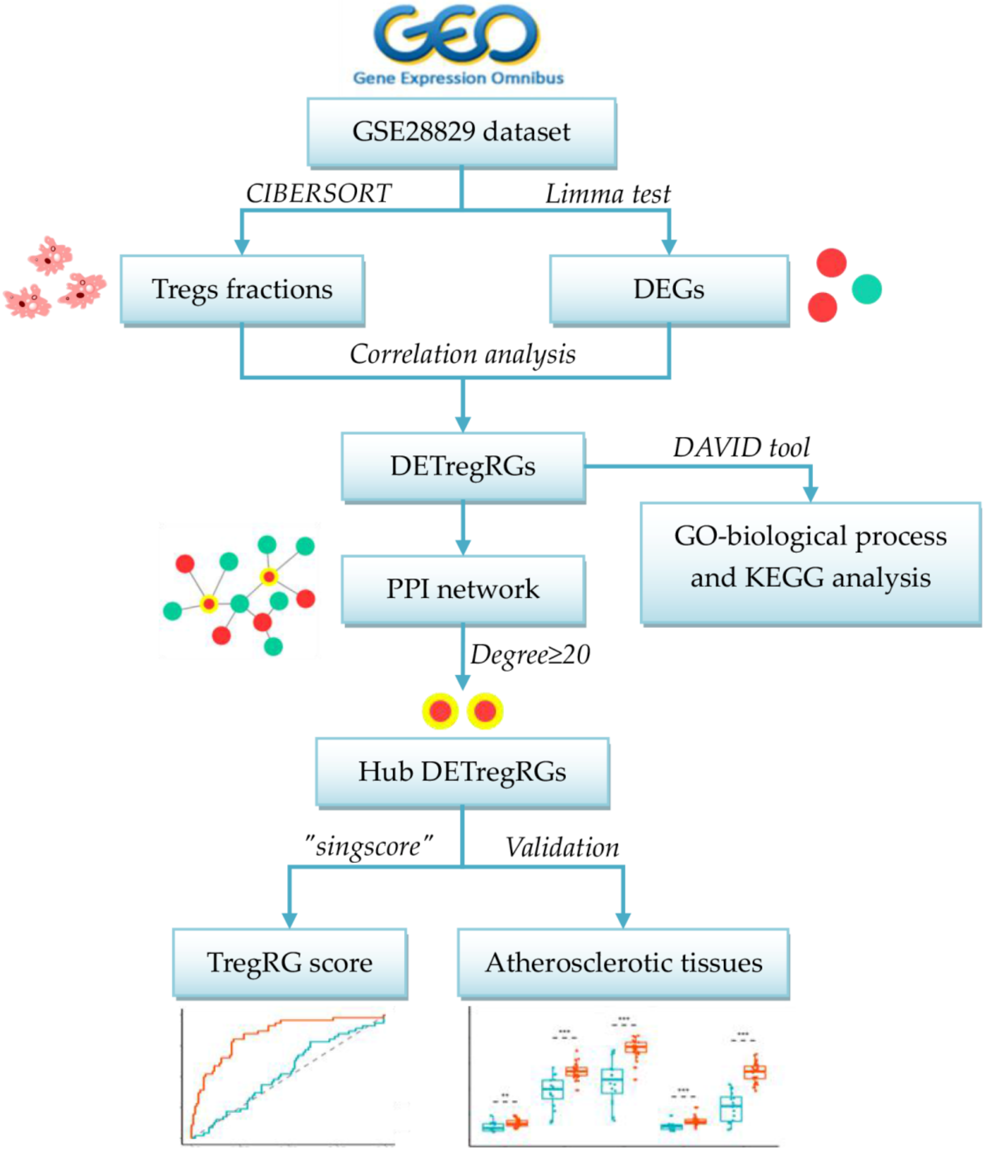

Abbreviations: DAVID, Database for Annotation, Visualization and Integrated Discovery; DEGs, differentially expressed genes; DETregRGs, differentially expressed Treg-related genes; GO, Gene Ontology; PPI, protein-protein network

Supplementary Table 1. The detailed information of the datasets used in this study

Accession ID	Database	Platform	Sample information; *URL*
**GSE28829**	GEO	Affymetrix Human Genome U133 Plus 2.0 Array	29 human carotid atherosclerotic plaques (16 at advanced stage and 13 at early stage); *https://www.ncbi.nlm.nih.gov/geo/query/acc.cgi?acc= GSE28829*
**GSE120521**	GEO	Illumina HiSeq 2500 (Homo sapiens)	8 human carotid atherosclerotic plaques (4 in stable and 4 in unstable regions); *https://www.ncbi.nlm.nih.gov/geo/query/acc.cgi?acc= GSE120521*
**E-TABM-190**	ArrayExpress	Affymetrix GeneChip Human Genome HG-U133A	11 human carotid atherosclerotic plaques (4 in stable and 7 in unstable regions); *https://www.ebi.ac.uk/arrayexpress/experiments/E-TABM-190/*
**GSE163154**	GEO	Illumina humanRef-8 v2.0 expression beadchip	43 human carotid atherosclerotic plaques (27 with and 16 without intraplaque haemorrhage); *https://www.ncbi.nlm.nih.gov/geo/query/acc.cgi?acc=GSE163154*
**GSE59867**	GEO	Affymetrix Human Gene 1.0 ST Array [transcript (gene) version]	157 peripheral blood samples collected at admission from patients with ST-segment elevation myocardial infarction (n=111) and patients with stable coronary artery disease (n=46); *https://www.ncbi.nlm.nih.gov/geo/query/acc.cgi?acc= GSE59867*
**GSE62646**	GEO	Affymetrix Human Gene 1.0 ST Array [transcript (gene) version]	42 peripheral blood mononuclear cell samples collected at admission from patients with ST-segment elevation myocardial infarction (n=28) and patients with stable coronary artery disease (n=14); *https://www.ncbi.nlm.nih.gov/geo/query/acc.cgi?acc= GSE62646*

Supplementary Table 2. The deconvolution p-value for each sample in GSE28829 dataset

Sample ID	p-value	Inclusion in analysis of immune cells
**GSM714070**	0	yes
**GSM714071**	0	yes
**GSM714072**	0	yes
**GSM714073**	0	yes
**GSM714074**	0	yes
**GSM714075**	0	yes
**GSM714076**	0.01	yes
**GSM714077**	0	yes
**GSM714078**	0	yes
**GSM714079**	0	yes
**GSM714080**	0	yes
**GSM714081**	0	yes
**GSM714082**	0	yes
**GSM714083**	0	yes
**GSM714084**	0	yes
**GSM714085**	0	yes
**GSM714086**	0.01	yes
**GSM714087**	0	yes
**GSM714088**	0.01	yes
**GSM714089**	0.01	yes
**GSM714090**	0.01	yes
**GSM714091**	0.26	no
**GSM714092**	0.04	yes
**GSM714093**	0	yes
**GSM714094**	0.01	yes
**GSM714095**	0.03	yes
**GSM714096**	0	yes
**GSM714097**	0.06	no
**GSM714098**	0.01	yes

Supplementary Table 3. The list of differentially expressed Tregs-related genes

Gene.symbol	Differentially expression analysis				Correlation analysis	
	logFC	AveExpr	t	p-value	PCC	p-value
**ADAP2**	1.2330	7.0974	8.0524	0.0000	-0.6166	0.0006
**VAMP8**	1.8254	7.5400	7.8869	0.0000	-0.6003	0.0009
**C3AR1**	1.5901	8.3272	7.7588	0.0000	-0.7030	0.0000
**SERPINA1**	0.9780	5.5265	7.7522	0.0000	-0.6093	0.0007
**AMPD3**	1.2538	6.7829	7.5183	0.0000	-0.6169	0.0006
**KCNE3**	0.5206	4.8178	7.1661	0.0000	-0.6718	0.0001
**FCGBP**	1.8263	8.0505	7.0809	0.0000	-0.6066	0.0008
**CD14**	1.6961	8.9846	6.9743	0.0000	-0.6051	0.0008
**CCR1**	1.5077	6.5829	6.9647	0.0000	-0.6425	0.0003
**C1QB**	1.9055	8.8463	6.9280	0.0000	-0.6554	0.0002
**MYO1F**	1.1116	6.6485	6.8865	0.0000	-0.6658	0.0002
**FERMT3**	1.1067	6.9794	6.8509	0.0000	-0.6487	0.0003
**RAC2**	1.1057	6.5419	6.5366	0.0000	-0.6858	0.0001
**FYB**	0.7592	5.2559	6.5004	0.0000	-0.6345	0.0004
**C1QA**	1.4529	8.9419	6.4769	0.0000	-0.6128	0.0007
**IGHA2**	2.1075	6.9115	6.4253	0.0000	-0.6461	0.0003
**STK10**	0.4862	5.6729	6.3804	0.0000	-0.6386	0.0003
**TLR2**	1.0731	7.8097	6.2608	0.0000	-0.6305	0.0004
**HCK**	1.4247	6.5316	6.2219	0.0000	-0.6210	0.0005
**CD53**	0.6514	5.7369	6.1815	0.0000	-0.6836	0.0001
**ZFYVE21**	-0.6362	8.3642	-6.1751	0.0000	0.6463	0.0003
**PID1**	-0.4309	6.2656	-6.1600	0.0000	0.6240	0.0005
**ABCA1**	0.5922	6.1763	6.1582	0.0000	-0.6783	0.0001
**PIP5K1B**	-0.6563	4.4878	-6.1141	0.0000	0.7078	0.0000
**ADGRA3**	-0.3219	4.2352	-6.1132	0.0000	0.6598	0.0002
**GMFG**	1.0553	6.9752	6.0475	0.0000	-0.6327	0.0004
**ARCN1**	-0.4007	9.7568	-5.9880	0.0000	0.6008	0.0009
**APBB1IP**	0.3807	5.3697	5.8871	0.0000	-0.6345	0.0004
**LHFPL2**	0.8135	8.0421	5.7929	0.0000	-0.6243	0.0005
**BCAT1**	1.1321	6.5577	5.6222	0.0000	-0.7499	0.0000
**DNAJB5**	-0.9063	6.4961	-5.4854	0.0000	0.6674	0.0001
**DAPK1**	0.5976	5.3979	5.4845	0.0000	-0.6030	0.0009
**PLA2G2A**	1.0590	11.8946	5.4832	0.0000	-0.6692	0.0001
**CTNNA1**	-0.2691	8.1764	-5.4774	0.0000	0.6065	0.0008
**CCL3L3**	1.6605	6.6111	5.4755	0.0000	-0.6001	0.0009
**VSIG4**	1.4312	9.1127	5.4719	0.0000	-0.6223	0.0005
**DNAJB6**	-0.5569	7.7305	-5.4328	0.0000	0.6231	0.0005
**MARCO**	1.2012	7.2569	5.4299	0.0000	-0.6135	0.0007
**NDRG3**	-0.6967	7.5387	-5.3876	0.0000	0.6289	0.0004
**MYCBP2**	0.5750	8.9467	5.3697	0.0000	-0.6038	0.0009
**MS4A4A**	1.5910	7.5150	5.3074	0.0000	-0.6068	0.0008
**MAFB**	0.9910	8.7587	5.2996	0.0000	-0.6026	0.0009
**NCF2**	1.5378	5.9474	5.2392	0.0000	-0.6851	0.0001
**FAM219B**	-0.4398	6.4338	-5.1808	0.0000	0.7539	0.0000
**STEAP1**	0.9416	7.6654	5.1729	0.0000	-0.6306	0.0004

**MTSS1**	0.2922	5.4295	5.1509	0.0000	-0.6852	0.0001
**MXRA7**	-0.5624	8.8286	-5.1087	0.0000	0.6113	0.0007
**LOC100129518**	0.8661	6.7671	5.0915	0.0000	-0.6962	0.0001
**LGALSL**	-0.4280	6.8907	-5.0908	0.0000	0.6835	0.0001
**PTPRC**	0.9694	5.2711	5.0688	0.0000	-0.6690	0.0001
**MAN2B2**	-0.3874	7.9169	-4.9760	0.0000	0.6117	0.0007
**CXCR4**	1.8139	8.4167	4.9609	0.0000	-0.6245	0.0005
**F11R**	0.5480	6.1119	4.9530	0.0000	-0.6003	0.0009
**PAG1**	0.7126	5.3883	4.9396	0.0000	-0.8125	0.0000
**ALOX5AP**	1.2945	8.3656	4.9165	0.0000	-0.6931	0.0001
**EVI2A**	1.4138	6.8988	4.7715	0.0000	-0.6625	0.0002
**TLR5**	0.8211	6.1229	4.7058	0.0001	-0.6633	0.0002
**PTPN11**	-0.3348	5.9563	-4.6871	0.0001	0.6155	0.0006
**RNASE6**	1.5328	6.9165	4.6568	0.0001	-0.6320	0.0004
**ROBO4**	0.4075	6.3145	4.5848	0.0001	-0.6146	0.0006
**HCLS1**	1.0591	8.0735	4.5472	0.0001	-0.6687	0.0001
**MYO18B**	-0.6050	5.1664	-4.4605	0.0001	0.6426	0.0003
**IL7R**	1.0088	5.8567	4.4441	0.0001	-0.6229	0.0005
**INPP5A**	-0.8645	7.6158	-4.3917	0.0001	0.6078	0.0008
**LYN**	0.9536	7.3214	4.3711	0.0001	-0.6793	0.0001
**CNTN1**	-0.7896	4.8551	-4.2918	0.0002	0.6621	0.0002
**THSD4**	-0.2588	5.0266	-4.2806	0.0002	0.6422	0.0003
**CCL5**	0.9950	6.5971	4.2661	0.0002	-0.6236	0.0005
**CMTM4**	-0.2544	5.2459	-4.2183	0.0002	0.6148	0.0006
**EVI2B**	1.6666	6.8520	4.2024	0.0002	-0.7307	0.0000
**NPR1**	-0.7038	8.4528	-4.1587	0.0002	0.6162	0.0006
**PDGFRA**	0.2083	6.0360	4.1291	0.0003	-0.6811	0.0001
**SPRYD7**	-0.2058	4.5060	-4.1267	0.0003	0.6646	0.0002
**NAP1L4**	-0.1382	5.5271	-4.1260	0.0003	0.6078	0.0008
**AFF3**	-0.1781	3.9423	-4.0852	0.0003	0.6136	0.0007
**CLSTN2**	0.8811	6.0368	4.0844	0.0003	-0.6097	0.0007
**CYTIP**	1.4208	6.2720	4.0633	0.0003	-0.6160	0.0006
**FABP3**	-0.6331	6.0235	-4.0009	0.0004	0.6073	0.0008
**BCL2A1**	1.4045	5.4616	3.9943	0.0004	-0.6433	0.0003
**ALDH1B1**	-0.8231	6.6825	-3.9923	0.0004	0.7419	0.0000
**MPP6**	-0.3644	3.7999	-3.9846	0.0004	0.6216	0.0005
**MKRN2**	-0.2165	5.8743	-3.9698	0.0004	0.6458	0.0003
**KCNK17**	-1.1680	7.8755	-3.9590	0.0004	0.6245	0.0005
**KCNT2**	1.3516	7.3153	3.9287	0.0005	-0.7162	0.0000
**ITGA9**	-0.3289	5.4836	-3.9116	0.0005	0.6625	0.0002
**SMPX**	-0.9192	4.9539	-3.8853	0.0005	0.7550	0.0000
**CENPV**	-0.4476	5.1136	-3.8752	0.0005	0.7697	0.0000
**GCHFR**	0.4080	5.9092	3.8546	0.0006	-0.6234	0.0005
**PDLIM7**	-0.5524	6.7796	-3.7862	0.0007	0.6179	0.0006
**PIAS3**	-0.3530	7.3549	-3.7820	0.0007	0.6309	0.0004
**EIF4H**	-0.2554	10.0561	-3.7554	0.0007	0.7291	0.0000
**ADCY5**	-0.4214	5.8618	-3.7520	0.0007	0.6655	0.0002
**LYST**	0.2814	5.4496	3.7188	0.0008	-0.6467	0.0003

**RAMP1**	-0.9702	8.5549	-3.6944	0.0009	0.6814	0.0001
**SLC2A12**	-0.4565	5.0143	-3.6913	0.0009	0.6478	0.0003
**CARD6**	0.7426	6.5927	3.6814	0.0009	-0.6669	0.0001
**DLG3**	-0.2037	5.7089	-3.6778	0.0009	0.7163	0.0000
**RAB11FIP1**	0.4670	6.7387	3.6618	0.0010	-0.6448	0.0003
**DEXI**	-0.3523	7.9176	-3.6454	0.0010	0.7246	0.0000
**LTBP4**	-0.4344	7.0407	-3.6382	0.0010	0.6168	0.0006
**ANO1**	-0.6448	7.0283	-3.6369	0.0010	0.6643	0.0002
**F13A1**	1.3499	9.0948	3.6096	0.0011	-0.6130	0.0007
**ELN**	-0.7076	8.3573	-3.6014	0.0011	0.6085	0.0008
**DYNLL2**	-0.1975	5.8934	-3.5563	0.0013	0.6029	0.0009
**GMPR**	-0.5873	6.8297	-3.5443	0.0013	0.8294	0.0000
**FAF1**	-0.2101	5.5809	-3.5433	0.0013	0.7595	0.0000
**PRPF40B**	-0.3025	5.7868	-3.5366	0.0013	0.6864	0.0001
**CFHR1**	0.5594	10.5889	3.5255	0.0014	-0.7080	0.0000
**LYNX1**	-0.3389	5.2689	-3.5108	0.0014	0.6254	0.0005
**RAB2A**	-0.3026	7.8087	-3.4807	0.0015	0.6554	0.0002
**TMEM98**	-0.3913	8.7875	-3.4754	0.0016	0.6166	0.0006
**N6AMT1**	-0.1497	4.5758	-3.4700	0.0016	0.6649	0.0002
**CMAHP**	0.3513	6.5721	3.4669	0.0016	-0.6900	0.0001
**GLRX**	0.6678	8.2848	3.4579	0.0016	-0.6040	0.0008
**ANKLE2**	-0.1756	5.1103	-3.4324	0.0018	0.6436	0.0003
**COL18A1**	-0.6528	8.4485	-3.4141	0.0018	0.6996	0.0000
**QSOX1**	-0.1994	5.5964	-3.3948	0.0019	0.7964	0.0000
**CARNS1**	-0.4495	6.2462	-3.3509	0.0022	0.6075	0.0008
**PHF2**	-0.1576	6.2064	-3.3164	0.0024	0.6164	0.0006
**PDZRN3-AS1**	-0.6264	5.0305	-3.2975	0.0025	0.6840	0.0001
**CFH**	0.7193	9.9407	3.2826	0.0026	-0.7158	0.0000
**UBA52**	0.2050	11.5835	3.2684	0.0027	-0.6822	0.0001
**SCUBE3**	-0.4509	6.8735	-3.2670	0.0027	0.6079	0.0008
**ZFAND3**	-0.2574	6.9574	-3.2616	0.0028	0.6605	0.0002
**VPS45**	-0.1951	6.2183	-3.2579	0.0028	0.6945	0.0001
**MTUS2**	-0.6034	6.0420	-3.2408	0.0029	0.6252	0.0005
**DAG1**	-0.3320	7.8293	-3.2287	0.0030	0.6111	0.0007
**MRC1**	1.2910	8.3681	3.1988	0.0032	-0.6019	0.0009
**ITPR2**	0.3306	5.2219	3.1856	0.0033	-0.6104	0.0007
**CD163**	1.0053	7.6675	3.1832	0.0034	-0.6033	0.0009
**CFHR2**	0.3456	6.4842	3.1743	0.0034	-0.6314	0.0004
**DCAF8**	-0.1094	5.0884	-3.1652	0.0035	0.6393	0.0003
**KIAA1551**	0.3801	5.7930	3.1603	0.0036	-0.6513	0.0002
**CLTB**	-0.1881	6.7444	-3.1397	0.0038	0.6558	0.0002
**TTLL11**	-0.2598	4.8258	-3.1359	0.0038	0.6408	0.0003
**MFAP4**	-0.7447	10.1354	-3.1163	0.0040	0.7207	0.0000
**SDCBP**	0.3938	11.4586	3.0922	0.0043	-0.6602	0.0002
**SYNRG**	0.1492	5.5952	3.0567	0.0047	-0.6380	0.0003
**CASQ1**	-0.3040	4.0261	-3.0501	0.0047	0.6133	0.0007
**C1QTNF2**	-0.6413	7.2622	-3.0448	0.0048	0.7249	0.0000
**PYGB**	-0.3305	6.2603	-3.0376	0.0049	0.7098	0.0000

**ASB1**	-0.2046	4.7045	-3.0017	0.0054	0.6001	0.0009
**LOC102723845**	-0.4534	5.0604	-2.9687	0.0058	0.7163	0.0000
**C15orf41**	-0.1104	4.5811	-2.9481	0.0061	0.6533	0.0002
**R3HCC1**	-0.2198	7.6213	-2.9336	0.0063	0.6791	0.0001
**PTGIR**	-0.3682	7.3437	-2.9281	0.0064	0.6954	0.0001
**ADGRE5**	-0.4637	9.1408	-2.9168	0.0066	0.6152	0.0006
**POLR2C**	-0.1599	7.1572	-2.9103	0.0067	0.7674	0.0000
**UHMK1**	0.3121	6.4168	2.9000	0.0069	-0.6183	0.0006
**LTBP3**	-0.2104	7.6476	-2.8569	0.0077	0.6799	0.0001
**BTBD10**	-0.1858	8.0028	-2.8410	0.0080	0.6424	0.0003
**ITM2C**	-0.6629	7.9500	-2.8272	0.0083	0.6914	0.0001
**OGDH**	-0.1580	5.6163	-2.8130	0.0086	0.6038	0.0009
**ACTR1B**	-0.2368	7.6086	-2.7990	0.0089	0.6216	0.0005
**SV2A**	-0.4664	7.1571	-2.7929	0.0090	0.6289	0.0004
**STRADB**	0.2623	6.7552	2.7898	0.0091	-0.6572	0.0002
**MYOZ1**	-0.4302	7.0702	-2.7603	0.0097	0.6264	0.0005
**BCL7B**	-0.2994	7.2257	-2.7525	0.0099	0.6507	0.0002
**DUSP27**	-0.6148	5.2964	-2.1112	0.0431	0.6928	0.0001

Supplementary Table 4. DETregRGs showing a PCC value >0.6 with each of the significant immune cells

Immune cell types	Count (%)	List of DETregRGs
**Naive B cells**	23 (14.5)	STEAP1, PTPRC, C1QB, CD14, C1QA, CXCR4, VAMP8, RNASE6, MS4A4A, RAB11FIP1, SERPINA1, HCK, TLR2, AMPD3, EVI2A, CD53, C3AR1, FCGBP, ALOX5AP, DCAF8, NPR1, KCNK17, and ADCY5
**Memory B cells**	2 (1.3)	KCNK17 and STEAP1
**Resting memory CD4$^+$ T cells**	2 (1.3)	SERPINA1 and MYO1F
**Gamma delta T cells**	2 (1.3)	NDRG3 and MFAP4
**Macrophages M0**	31 (19.5)	DNAJB6, DLG3, ADCY5, RAMP1, MYO18B, NPR1, CENPV, ITGA9, ZFYVE21, AMPD3, RNASE6, CYTIP, CD53, LHFPL2, HCLS1, HCK, FERMT3, CXCR4, TLR2, APBB1IP, MYO1F, BCAT1, CCL3L3, NCF2, GMFG, FYB, IL7R, RAC2, C1QA, ABCA1, and ALOX5AP
**Macrophages M2**	10 (6.3)	DCAF8, CASQ1, MYO18B, CLSTN2, MYCBP2, ROBO4, VAMP8, FCGBP, DAPK1, and LOC100129518
**Activated dendritic cells**	1 (0.1)	MAFB
**Resting mast cells**	0 (0.0)	None

Supplementary Table 5. The detailed steps (or R script) for data analyses

Step 1: Estimation of 22 immune cells for the atherosclerotic plaques (GSE28829 dataset)

```
source("immune.CIBERSORT.R")   ##the R script to run CIBERSORT that was downloaded from the CIBERSORT website
results<-CIBERSORT("ref.txt","Expr_GSE28829.txt", perm=100, QN=T)   ##Expr_GSE28829 is the gene expression matrix of GSE28829
results<-data.frame(results)   ## Supplementary Table 2
immune_data<-results[results$P.value<0.05,1:22]   ##removing samples with deconvolution p-value<0.05
group.info<-read.delim("clipboard",row.names = 1)   ##load the group information of GSE28829
group.info$group<-factor(group.info$group,levels=c("Early_plaque","Advanced_plaque"))
immune_data$group<-group.info[rownames(immune_data),]$group

######plot Figure 1A
library(ggplot2)
library(reshape2)
immune_boxplot<-cbind(sampleID=rownames(immune_data),immune_data[,1:22])
immune_boxplot<-melt(immune_boxplot,id.vars="sampleID")   ##create the input table for ggplot
names(immune_boxplot)[2:3]<-c("immune.cell","fraction")
ggplot(immune_boxplot,aes(x=sampleID,y=fraction,fill=immune.cell))+geom_bar(stat="identity")+theme_bw()
```

Step 2: Comparison of immune cell fractions between early and advanced atherosclerotic plaques (GSE28829 dataset)

```
stata.immune<-data.frame(immune.cell=rep("A",22),m.early=1:22,m.advanced=1:22,w.value=1:22,p.value=1:22,stringsAsFactors=F)   ##create a data frame to deposit the results of Wilcoxon rank sum test
for (i in 1:22) {
  groupmedian=aggregate(immune_data[,i],list(immune_data$Group),median)
  forma<-as.formula(paste(names(immune_data)[i],"Group",sep="~"))
  stata.W<-wilcox.test(forma,data=immune_data,paired = F)   ##Wilcoxon rank sum test
  stata.immune[i,1]<-names(immune_data)[i]
  stata.immune[i,2:3]<-groupmedian[,2]
  stata.immune[i,4:5]<-c(stata.W$statistic,stata.W$p.value)
}
```

```
######plot Figure 1B
library(ggpubr)
ggboxplot(immune_data,x="Group",y="Treg.cells",color="Group",add="jitter",ggtheme=theme_bw())
```

Step 3: Identification of differentially expressed genes (DEGs) between early and advanced plaques using limma test (GSE28829 dataset)

```
library(limma)
Expr<-read.table("Expr_GSE28829.txt",header=T,sep="\t",row.names = 1)
design<-model.matrix(~0+group.info$group)
colnames(design)<-c("Early_plaque","Advanced_plaque")
fit<-lmFit(Expr,design)
cont.matrix<-makeContrasts(Advanced_plaque-Early_plaque,levels=design)
fit1<-eBayes(contrasts.fit(fit,cont.matrix))
allDiff=topTable(fit1,adjust='fdr',number=200000)
logFoldChange=log2(1.5)   ##set the threshold for significance
Pvalue=0.01
diffSig<-allDiff[with(allDiff,(abs(logFC)>logFoldChange | P.Value<Pvalue )),]
```

Step 4: Identification of Tregs-related DEGs (DETregRGs) (GSE28829 dataset)

```
Expr_immune<-Expr[,rownames(immune_data)]
stata.corr<-data.frame(geneNames=rep("A",nrow(Expr_immune)),PCC.value=1:nrow(Expr_immune),p.value=1:nrow(Expr_immune),stringsAsFactor
s=F) ##create a data frame to deposit the results of Pearson correlation test
for (i in 1:nrow(Expr_immune)) {
  pcctest<-cor.test(as.numeric(Expr_immune[i,]),immune_data$Treg.cells) ##Pearson correlation analysis
  stata.corr[i,1]<-rownames(Expr_immune)[i]
  stata.corr[i,2:3]<-c(pcctest$estimate,pcctest$p.value)
}
rownames(stata.corr)<-stata.corr[,1]
stata.corr<-stata.corr[,-1]
```

```
diffSig_Tregcorr<-cbind(diffSig,stata.corr[rownames(diffSig),])
Treg_siggene_matrix<-subset(diffSig_Tregcorr,abs(PCC.value)>=0.6) ##DEGs with |PCC.value|>=0.6 were considered as DETregRGs
write.table(Treg_siggene_matrix,"DETregRGs_matrix.txt",sep="\t",quote = F,row.names = T,col.names = T) ###Supplementary Table 3

######plot Figure 1C
diffSig_Tregcorr$Group<-rep("Control",nrow(diffSig_Tregcorr))
diffSig_Tregcorr$Group[with(diffSig_Tregcorr,PCC.value>=0.6)]="Positive TregRGs"
diffSig_Tregcorr$Group[with(diffSig_Tregcorr,PCC.value<=-0.6)]="Negative TregRGs" ##create the input table for ggplot
ggplot(diffSig_Tregcorr,aes(x=logFC,y=abs(PCC.value)))+geom_point(aes(col=Group))+theme_bw()

####Clarify the associations between other significant immune cells and DETregRGs (Supplementary Table 4)
AA=data.frame(t(Expr_immune[rownames(Treg_siggene_matrix),]))
BB=immune_data[,stata.immune[stata.immune$p.value<0.05,]$immune.cell[-c(3,5,11)]]
otherimmune_corr<-data.frame(WGCNA::cor(AA,BB,use="pairwise.complete.obs"))
write.table(otherimmune_corr,"otherimmune_corr.txt",sep="\t",quote = F,row.names = T,col.names = T)
```

Step 5: Functional enrichment analysis of DETregRGs

######The list of 159 DETregRGs was uploaded into the DAVID 6.8 webpage (https://david.ncifcrf.gov/). We focused on functional annotations for Gene Ontology (GO)-biological process and Kyoto Encyclopedia of Genes and Genomes (KEGG) pathway. A functional annotation chart was downloaded from the result page and was then processed with the following R script:

```
Funchart<-read.table("Functional_chart.txt",header=T,sep="\t",stringsAsFactors=F)
Sig_ Funchart<-subset(Funchart, PValue<0.05 & length(Genes)>3) ##The significance threshold was set as p-value <0.05 and gene count>3; Table 1.
```

Step 6: Constriction of protein-protein network and identification of hub genes

######The list of 159 DETregRGs was uploaded into the STRING database (https://string-db.org) to obtain significant PPIs with combined scores >0.4. The PPIs in the csv format file downloaded from the result page were visualized using Cytoscape 3.6.0 software (**Figure 2**). The topological features of the PPI network were exported using the "NetworkAnalyzer" tool in Cytoscape and then processed with the following R script to identify hub DETregRGs:

```
Topoppi<-read.table("Topo_features.txt",header=T,sep="\t",stringsAsFactors=F)
```

```
Hubgenes<- as.vector(subset(Topoppi [order(Topoppi $Degree,decreasing=T),],Degree>=20)$name) ##gene nodes with degree>=20 were considered as hub DETregRGs
```

Step 7: Validation of hub genes in atherosclerotic plaques obtained from independent datasets (GSE120521, E-TABM-190, and GSE163154)

```
######plot Figure 3A (GSE28829 dataset)
library(pheatmap)
heat_data<-t(scale(t(Expr[Hubgenes,]))) ##Z-score transformation of the expressions of hub genes
anno_col<-data.frame(Group=group.info$group)
rownames(anno_col)<-rownames(group.info)
ann_color=list(Group=c(Early_plaque="#00AFBB",Advanced_plaque="#FC4E07"))     ##Annotation for early and advanced plaques
pheatmap(heat_data, annotation_col=anno_col,annotation_colors=ann_color,clustering_method = "complete",show_colnames = F,cluster_rows = F)

######plot Figure 3B (GSE120521 and E-TABM-190 datasets)
###limma test in GSE120521
Expr_GSE120521<-read.delim("clipboard",header=T)    ##read the RNA-seq data of GSE120521 ("GSE120521_Athero_RNAseq_FPKM.xlsx")
Expr_GSE120521<-Expr_GSE120521[Expr_GSE120521$name %in% Hubgenes,]
rownames(Expr_GSE120521)=Expr_GSE120521[,1]
Expr_GSE120521<-Expr_GSE120521[,-1]
Expr_GSE120521<-log2(Expr_GSE120521+1)    ##log2(FPKM+1) transformation
Group_GSE120521<-rep(c("con","unstable"),5)     ##create group information of GSE120521
design<-model.matrix(~0+factor(Group_GSE120521))
colnames(design)<-c("con","unstable")
fit<- lmFit(Expr_GSE120521,design)
cont.matrix<-makeContrasts(unstable-con,levels=design)
fit2<-contrasts.fit(fit, cont.matrix)
fit2<-eBayes(fit2)
allDiff_GSE120521=topTable(fit2,adjust='fdr',number=200000)
###read the results of limma test of E-TABM-190 that can be directly downloaded from ArrayExpress ("E-TABM-190.processed.1.zip").
allDiff_ETABM190<-read.delim("clipboard",header=T)
```

```
hub_ETABM190<-subset(Expr_ETABM190,geneSymbol==Hubgenes)
###prepare the input file for plotting Figure 3B
hub_GSE120521<-allDiff_GSE120521[Hubgenes,]
allDiff_GSE120521<-allDiff_GSE120521[,c(1,4)] ##select logFC and P.value columns
allDiff_GSE120521$genesymbol<-rownames(allDiff_GSE120521)
allDiff_GSE120521<-allDiff_GSE120521[,c(3,1,2)]
rownames(allDiff_GSE120521)=NULL
plauqe_validata<-rbind(hub_GSE120521, hub_ETABM190)
plauqe_validata$datasets<-factor(c(rep("GSE120521",5),rep("E-TABM-190",5)),levels=c("GSE120521","E-TABM-190"))
plauqe_validata$logFC<-as.numeric(plauqe_validata$logFC)
plauqe_validata$P.Value<-as.numeric(plauqe_validata$P.Value)
plauqe_validata$Pcut<-factor(cut(plauqe_validata$P.Value,breaks=c(0,0.001,0.01,0.05,1),labels=c("P<0.001","P<0.01","P<0.05","P⩾0.05")),
                              levels=rev(c("P<0.001","P<0.01","P<0.05","P⩾0.05")))
ggplot(plauqe_validata,aes(genesymbol,datasets))+geom_point(aes(size=Pcut,color=logFC))

######plot Figure 3C (GSE163154 dataset)
Expr_GSE163154<-read.table("Expr_GSE163154.txt",header=T,sep="\t",row.names = 1) ##Expr_GSE163154 is the gene expression matrix of GSE163154
Group_GSE163154<-read.delim("clipboard",header=T,row.names = 1) ##read the group information of GSE163154
Expr_GSE163154<-data.frame(t(Expr_GSE163154[Treg.genes,]))
identical(rownames(Expr_GSE163154),rownames(Group_GSE163154))
Expr_GSE163154$Group<-Group_GSE163154$Group
Wstata_GSE163154<-data.frame(Genes=rep("A",5),W.value=1:5,p.value=1:5,stringsAsFactors=F)    ##create a data frame to deposit the results of Wilcoxon rank sum test
for (i in 1:5) {
  forma<-as.formula(paste(names(Expr_GSE163154)[i],"Group",sep="~"))
  stata.W<-wilcox.test(forma,data=Expr_GSE163154,paired=F)   ##Wilcoxon rank sum test between plaques with and without IPH
  Wstata_GSE163154[i,1]<-names(Expr_GSE163154)[i]
  Wstata_GSE163154[i,2:3]<-c(stata.W$statistic,stata.W$p.value)
}
```

```
Wsata_plotdata<-melt(Expr_GSE163154,id.vars="Group")
names(Wsata_plotdata)[2:3]<-c("geneNames","geneExp")
ggboxplot(Wsata_plotdata,x="geneNames",y="geneExp",color="Group",add="jitter", ggtheme=theme_bw())
```

Step 8: Construction of hub DETregRGs signature for discriminating MI patients (GSE59867 and GSE62646)

```
library(singscore)
library(pROC)
###Construction of hub DETregRGs signature by "singscore" method in GSE59867 dataset
Expr_GSE59867<-read.table("Expr_GSE59867.txt",header = T,row.names = 1) ##Expr_GSE59867 is the gene expression matrix of GSE59867
Group_GSE59867<-read.table("Group.txt",stringsAsFactors = F,header=T,row.names = 1,sep="\t") ##read the group information of GSE59867
Group_GSE59867<-subset(Group_GSE59867,Group %in% c("on the 1st day of MI (admission)", "sCAD")) ##remove samples not collected at admission
Expr_GSE59867<- Expr_GSE59867[,rownames(Group_GSE59867)]
rankData_GSE59867<-rankGenes(Expr_GSE59867)
sscore_GSE59867<-simpleScore(rankData_GSE59867, upSet=Hubgenes,knownDirection = F)
Group_GSE59867$TregRGscore<-as.numeric(sscore_GSE59867[,1])
Group_GSE59867$Group<-factor(Group_GSE59867$Group,levels = c("sCAD","on the 1st day of MI (admission)"),labels = c("sCAD","MI"))
roc_GSE59867<-roc(Group_GSE59867$Group,Group_GSE59867$TregRGscore)##ROC analysis
auc(roc_GSE59867)
##Construction of random signature for comparison using Bootstrap method
set.seed(20210301)
random.gene<-sample(rownames(data_GSE59867),5,replace=F)
sscore_GSE59867.random<-simpleScore(rankData_GSE59867, upSet=random.gene, knownDirection = F)
roc_GSE59867.random<-roc(Group_GSE59867$Group,as.numeric(sscore_GSE59867.random[,1]))
roc.test(roc_GSE59867,roc_GSE59867.random,method="bootstrap")

###Construction of hub DETregRGs signature by "singscore" method in GSE62646 dataset
Expr_GSE62646 <-read.table("Expr_GSE62646.txt",header = T,row.names = 1) ##Expr_GSE62646 is the gene expression matrix of GSE62646
Group_GSE62646<-read.table("Group.txt",stringsAsFactors = F,header=T,row.names = 1,sep="\t") ##read the group information of GSE62646
```

```
Group_GSE62646<-subset(Group_GSE62646,Time=="admission")##remove samples not collected at admission
Expr_GSE62646<- Expr_GSE62646[,rownames(Group_GSE62646)]
rankData_GSE62646<-rankGenes(Expr_GSE62646)
sscore_GSE62646<-simpleScore(rankData_GSE62646, upSet=Hubgenes,knownDirection = F)
Group_GSE62646$TregRGscore<-as.numeric(sscore_GSE62646[,1])
Group_GSE62646$Group<-factor(Group_GSE62646$Group,labels=c("sCAD","MI"))
roc_GSE62646<-roc(Group_GSE62646$Group,Group_GSE62646$TregRGscore)##ROC analysis
auc(roc_GSE62646)
##Construction of random signature for comparison using Bootstrap method
set.seed(20210324)
random.gene1<-sample(rownames(data_GSE62646),5,replace=F)
sscore_GSE62646.random<-simpleScore(rankData_GSE62646, upSet=random.gene1, knownDirection = F)
roc_GSE62646.random<-roc(Group_GSE62646$Group,as.numeric(sscore_GSE62646.random[,1]))
roc.test(roc_GSE62646,roc_GSE62646.random,method="bootstrap")

###plot Figure 4A
wilcox.test(TregRGscore~Group,data=Group_GSE59867)
ggboxplot(Group_GSE59867,x="Group",y="TregRGscore",color="Group",add="jitter",ggtheme=theme_bw())
###plot Figure 4B
wilcox.test(TregRGscore~Group,data=Group_GSE62646)
ggboxplot(Group_GSE62646,x="Group",y="TregRGscore",color="Group",add="jitter",ggtheme=theme_bw())
###plot Figure 4C
ggroc(list(TregRG.Score=roc_GSE59867,Random=roc_GSE59867.random)+geom_line(size=1.2)+
annotate("segment",x=1,xend=0,y=0,yend=1,col="gray50",size=0.9,linetype=2)+theme_bw()
###plot Figure 4D
ggroc(list(TregRG.Score=roc_GSE62646,Random=roc_GSE62646.random)+geom_line(size=1.2)+
annotate("segment",x=1,xend=0,y=0,yend=1,col="gray50",size=0.9,linetype=2)+theme_bw()
```
